# Associations of Empathy with Teacher–Student Interactions: A Potential Ternary Model

**DOI:** 10.3390/brainsci13050767

**Published:** 2023-05-06

**Authors:** Binghai Sun, Yaoyao Wang, Qun Ye, Yafeng Pan

**Affiliations:** 1 Intelligent Laboratory of Child and Adolescent Mental Health and Crisis Intervention of Zhejiang Province, School of Psychology, Zhejiang Normal University, Jinhua 321004, China; 2 Key Laboratory of Intelligent Education Technology and Application of Zhejiang Province, Zhejiang Normal University, Jinhua 321004, China; 3 Department of Psychology and Behavioral Sciences, Zhejiang University, Hangzhou 310058, China

**Keywords:** interpersonal neuroscience, teacher empathy, teacher–student interaction, education, hyperscanning

## Abstract

Empathy has garnered increasing recognition as a pivotal component of teacher–student interactions and a notable determinant of student achievement. Nevertheless, the exact impact of empathy on teacher–student interactions remains elusive, despite research endeavors into the neural mechanisms of teacher empathy. Our article examines the cognitive neural processes of teacher empathy during various forms of teacher–student interactions. To this end, we first present a concise review of theoretical considerations related to empathy and interactions, followed by an extensive discussion of teacher–student interactions and teacher empathy through both “single-brain” and “dual-brain” perspectives. Drawing on these discussions, we propose a potential model of empathy that integrates the affective contagion, cognitive evaluation, and behavior prediction aspects of teacher–student interactions. Finally, future research directions are discussed.

## 1. Introduction

Teaching and learning are highly social. Within the education setting, teacher–student interaction stands out as one of the most fundamental and necessary form of interpersonal communication [1]. Teacher–student interaction refers to the various forms, natures, and degrees of engagement that occur between teachers and students [2]. This interaction not only triggers students’ cognitive development and social growth but also benefits teachers’ professional growth [2,3,4]. The quality of teacher–student interactions depends on their ability to understand each other’s intentions and emotions (i.e., empathy).

Teacher empathy is the ability of a teacher to understand and share the feelings and perspectives of their students [5]. This includes the teacher’s capability to recognize and address their students’ emotional needs, and adjust their teaching and guidance accordingly. Teachers who exhibit empathy are better equipped to discern a student’s negative emotions, such as sadness over a poor grade or boredom with a particular learning task, from their facial expressions [6]. Creating a positive emotional atmosphere in the classroom, responding sensitively to the emotional, social, and academic needs of students, and taking into account their interests are all components of high-quality interactions that contribute to teachers’ ability to provide emotional support and manage their classrooms effectively [7]. Therefore, teacher empathy is a key factor shaping the quality of teacher–student interactions and cultivating positive teacher–student relationships.

Although recent research has explored the link between empathy and teacher–student interactions, there are some limitations when it comes to explaining their underlying mechanisms and research methodology. It is unclear how empathy differs from other psychological factors. While some researchers define empathy as the ability to understand other’s emotional experiences [8], independent of theory of mind (ToM), others argue that empathy and ToM share common brain regions [9]. For this study, in accordance with Baron-Cohen and Wheelwright, we define empathy as including both affective and cognitive components: affective empathy, which describes the ability to vicariously share a target’s internal state, and cognitive empathy, which refers to the understanding of target’s internal states without sharing them [10].

In addition, by relying solely on questionnaires and behavioral experiments, traditional research on teacher empathy has provided limited understanding of the cognitive and neural mechanisms involved, and how they impact behavior. However, with the use of neuroscience technologies such as electroencephalography (EEG) and functional near-infrared spectroscopy (fNIRS), researchers are now able to directly observe brain activity [11,12,13], providing more immediate and objective physiological evidence. This approach can further reveal the cognitive neural mechanisms involved in teacher–student interaction, and enhance our understanding of teacher empathy [14,15,16,17,18].

This paper aims to expand our understanding of how teacher empathy influences teacher–student interactions. This will be achieved by an analysis of the possible mechanisms that teacher empathy employs to affect these interactions. A potential ternary model of empathy during teacher–student interaction will be proposed, which will offer theoretical guidance for further research in this area. Ultimately, this review aims to emphasize the significance of teacher empathy and encourage its incorporation into the educational context.

## 2. Theoretical Considerations for Empathy and Interpersonal Interaction

To understand how teacher empathy impacts teacher–student interactions, we first scrutinized past theoretical considerations pertaining to empathy and general interactions. General interactions refer to the overall communication and behavior between individuals or groups in a social encounter [19,20]. Our analysis centered on four broad strains of models, namely the shared intentionality theory, theory of mind, the perception–action model, and perspective taking.

*Shared Intentionality Theory* is a psychological framework that elucidates how people can effectively collaborate and coordinate their actions towards a common goal [21]. This theory posits that individuals can synchronize their behavior and interact more effectively by sharing mental states such as intentions, beliefs, and desires. Research has shown that interpersonal synchronization based on shared intentions can substantially enhance cooperative behavior [22]. When teachers possess high levels of empathy, they endeavor to align their conduct with that of their students, which results in an improved quality of teacher–student interactions and greater efficacy of the teaching process.

*Theory of Mind (ToM)* pertains to the ability to grasp and deduce both one’s own and others’ mental states, and to use this information to predict and clarify their actions [23]. ToM and empathy are key aspects of interpersonal communication, allowing individuals to perceive and understand the actions of others. Although certain research suggests that empathy does not necessarily require ToM [8], others propose that empathy and ToM share common brain regions [23]. ToM involves the cognitive process of recognizing and evaluating the emotions of others, while empathy involves the capacity to understand the emotional experiences of others [24].

*Perception–Action Model (PAM)* proposes that empathy is a product of the evolutionary development of species, as demonstrated by the ability of non-human social mammals such as apes and mice to imitate others [25]. This imitation helps individuals to learn and respond appropriately in dangerous situations, and encourages pro-social behavior among peers. The PAM suggests that when an individual observes the behavior of others, they will create a mental representation of their own experience with that behavior, and simultaneously imitate it.

*Perspective Taking (PT)* is the ability to distinguish between one’s own viewpoint from that of others, and to infer and react to the perspectives of others using relevant information [26]. It has been suggested that this essential aspect of empathy is crucial for teachers, and has a significant impact on the teacher–student relationship and students’ academic performance [5].

To summarize, it can be inferred that empathy is a multifaceted psychological process that encompasses cognitive, emotional, and behavioral factors. However, none of the presented theoretical considerations offered a complete understanding of teacher empathy in the context of teacher–student interactions. The PAM proposes that empathy arises from passive and automatic imitation, while the ToM and PT models concentrate primarily on the cognitive aspect and do not fully address the emotional aspect. Furthermore, in teacher–student interactions, it is insufficient for teachers to only understand and empathize with their students’ emotions, and they must also demonstrate appropriate behavioral reactions to them.

## 3. The Impact of Empathy on Teacher–Student Interaction: Single-Brain Findings

Echoing the theories previously discussed, recent years have also seen the use of advanced neuroimaging tools to provide neural evidence for empathy and teacher–student interaction.

The mirror neuron system (MNS) has been discovered and is believed to support the PAM theory, as it activates the same regions of the brain when people observe others’ actions as when they perform the actions themselves [27]. Affective empathy involves the MNS, specifically the inferior frontal gyrus (IFG), as well as the insula, anterior cingulate gyrus, and limbic system [28,29,30]. While MNS is likely to mediate the cognitive processes underlying interpersonal synchrony, it only provides a low-level mechanism for matching the self-state with the target state, mainly involving the motor system [31].

The brain region responsible for ToM is more strongly associated with cognitive empathy. The medial prefrontal cortex (mPFC), superior temporal sulcus (STS), temporoparietal junction (TPJ), and temporal pole (TP) are neural networks engaged in ToM and PT, while the ventromedial prefrontal cortex (vmPFC) is a crucial brain region for cognitive empathy [32,33]. Despite their relationship, ToM and cognitive empathy are distinct concepts: ToM primarily involves cognitive control processing, whereas cognitive empathy involves both controlled cognitive processing and automatic processing, including emotional processing in the anterior cingulate cortex (ACC), amygdala, and vmPFC [34]. The STS is a crucial component of ToM, as it allows teachers to make inferences about their students’ understanding and adjust their teaching approach accordingly. Furthermore, shared intentionality is closely related to cognitive empathy. Studies have revealed that the prefrontal cortex (PFC) plays a crucial role in shared intentionality, necessitating that individuals consciously synchronize their thoughts with others to enhance their collaborative behavior [35].

In brief, effective communication between teachers and students relies on both affective empathy and cognitive empathy, which activate different neural networks in the brain. Affective empathy involves the anterior insula (AI) and ACC, the mirror neuron system (MNS), while cognitive empathy is associated with brain regions similar to ToM, PT (PFC, in particular) and shared intentionality. Although research indicates that teacher empathy significantly impacts teacher–student interaction and is linked with the MNS, ToM, and other brain regions, there is still no systematic analysis of the mechanism of teacher empathy in the interaction. It remains unclear whether effective communication between teachers and students primarily relies on the MNS, PFC, or the coordination of various neural systems, and whether these factors apply to different aspects of teacher–student interaction.

To clarify these issues, a number of researchers have examined the impact of the teacher empathy on teacher–student interactions from a single-brain perspective. Takeuchi and co-workers used fNIRS to investigate how teachers and students collaborated when solving puzzles [36]. The results showed a significant increase in the activation of the PFC of teachers after providing prompts, which suggested that teachers deliberately understood their students’ responses during the interaction. Furthermore, the personality traits of teachers can affect the level of empathy present in teacher–student interactions. Using EEG, Zhu and colleagues investigated the neurocognitive mechanisms of pain empathy in teachers with strong or weak professional identities [37]. The results demonstrated that teachers with a strong professional identity had a substantial increase in N110 amplitude, which is an essential indicator of emotional sharing in pain empathy. This suggests that professional teachers are more adept at sharing emotions with students. In addition, a separate study has revealed that teachers with high emotional awareness abilities were able to rapidly perceive their students’ emotional cues and express emotions, which led to an increase in the amplitudes of N100, P100, and N170 [38]. These teachers also had the ability to identify their students’ emotional types, resulting in a rise in the amplitude of late positive potential (LPP) [38].

Unfortunately, conventional neuroimaging studies primarily focus on individual neurocognitive reactions and only measure the teacher’s (or student’s) brain activity in response to social situations, which makes it difficult to understand the complete brain mechanism that underlies the entire teacher–student interaction.

## 4. Findings from Dual-Brain (Hyperscanning) Studies

To surmount this constraint, studies using e.g., EEG and fNIRS have been integrated with the hyperscanning technique; these studies focus on the interdependencies of the brain activity of two or more individuals during an interaction, employing interpersonal brain synchronization (IBS) as a gauge of the interaction [39,40,41]. This article categorizes current hyperscanning research of teacher–student interaction into three groups: (1) the observation of perception and movement, (2) the assessment of information transmission and processing, and (3) the exploration of the collaborative process [42].

### 4.1. The Observation of Perception and Movement

Research in this particular area centers on examining the brain’s functioning during the execution of a specific task, such as tapping to a rhythm or mimicking an action [43,44,45], without requiring complex cognitive processes such as thinking or decision-making. These types of tasks activate neural networks such as the MNS, sensorimotor area, and PFC [46,47], which are associated with the perceptual-motor aspects of social interaction.

As previously mentioned, the concepts of empathy and interpersonal synchrony have been linked to the activation of embodied representations in the brain through a perception–action mechanism, involving the simulation of observed actions and emotions [48]. Studies have shown that individuals high in empathy tend to anticipate the actions of others during joint musical activities, resulting in better synchronization with their partners [49]. This idea is supported by the shared affective motion experience (SAME) model proposed by Overy et al., which suggests that the MNS is involved in perceiving and understanding both actions and emotions conveyed through music [50].

In contrast to music, the teacher–student interaction that involves perception and movement requires cognitive engagement. Xu used fNIRS hyperscanning to examine the impact of imitation on intention understanding [51]. In this study, 34 teachers (half of whom were experts and half of whom were novices) were instructed to imitate their students’ gestures and then predict their intentions. The researchers assessed the teachers’ empathic abilities and ToM. Interestingly, novice teachers showed significantly greater PT ability (the cognitive component of empathy) than expert teachers. Additionally, the fNIRS results revealed that the novice teacher–student pairs had a significantly higher degree of IBS in the dorsolateral prefrontal cortex (DLPFC) compared the expert teacher–student pairs. This finding could be explained by the smaller age gap between novice teachers and their students, which might have facilitated better communication and understanding between them, ultimately leading to improved learning outcomes. Another study conducted by Xu and colleagues [51], in which expert and novice teachers were asked to imitate their students’ drawings and independently assess their intentions, also discovered higher IBS in novice teacher–student pairs compared to expert-student pairs. The process of imitation entails not only reproduction of the external behavior of the imitated person by the imitator, but also intricate internal reasoning. As a result, when teachers imitate their students’ movements, they must infer their intentions, which necessitates cognitive activity.

### 4.2. The Assessment of Information Transmission and Processing

In the context of teacher–student interactions, both verbal communication and eye contact are often used to convey thoughts and emotions. The Heuristic working model suggests that teacher empathy is crucial in providing emotional support [52], which is a key aspect of high-quality teacher–student interactions. Several studies have indicated that teachers with higher levels of empathy tend to better perceive others’ emotions and consider students’ needs, leading to the formation of positive relationships. Teachers with greater empathy are more likely to adopt emotionally supportive strategies, such as comfort and encouragement, when dealing with challenging student behaviors [3]. Additionally, the quality of the teacher–student relationship has been found to affect teacher–student synchronization [18]. Students who felt socially closer to their teacher showed higher levels of IBS with them. Since IBS may serve as an indicator of attentional engagement [12], good teacher–student relationships can lead to increased student engagement in class.

Additionally, teacher empathy can play a role in the support provided during instructional interactions. By demonstrating empathy, teachers can provide feedback and foster classroom discussions related to the teaching content. To effectively tailor their teaching to meet the needs of learners and create engaging lessons, teachers must be able to identify when students are struggling to understand content and which activities they find appealing or dull [53,54]. Researchers have explored the relationship between teachers’ empathy and instructional support by using self-report questionnaires completed by teachers and students. The findings indicate a positive correlation between emotional intelligence and teaching effectiveness, with teachers who exhibit higher emotional intelligence being more efficient in providing instructional support [7]. Additionally, students reported that teachers who demonstrated a greater degree of perceived emotions were more organized and provided clearer instruction [55].

According to the “prediction–transmission” hypothesis, in order to provide effective instructional support, teachers must accurately predict the learning status of their students [15]. In a study conducted by Zheng et al., the brain activity of both teachers and students was measured simultaneously, and it was found that better teaching outcomes were associated with higher levels of IBS between the right TPJ of the teacher and the anterior superior temporal cortex (aSTC) of the student, when the teacher’s brain activity preceded that of the student. The TPJ has been identified as a crucial area for ToM, and as the cognitive component of empathy in previous studies [56]. Therefore, successful teacher–student interactions may require teachers to speculate about the mental states of their students, indicating that a teacher’s (cognitive) empathy is a crucial factor in achieving positive outcomes in these interactions.

### 4.3. The Exploration of the Collaborative Process

Another strain of research on teacher–student interaction has focused on collaborative processes. Such investigations entail scrutinizing brain functions associated with motivation and social decision-making, and contrasting the neural activities of individuals when performing cooperative tasks [11,41]. The cingulate gyrus, TPJ, and PFC are among the brain regions that have been linked to these cognitive processes [57,58,59,60,61,62].

Previous studies have revealed that empathy plays a pivotal role in cooperative tasks; individuals who possessed a greater empathetic ability performed better in collaborative endeavors [63]. An individual’s empathic capacity affected the joint Simon effect, which was amplified when interacting with a friend [63]. It is plausible that individuals with higher levels of empathy are more attuned to the emotions and requirements of others during social interactions, thereby facilitating the harmonious flow of the collaborative process [64].

Through hyperscanning techniques, some researchers have delved into the neural mechanism of empathy during cooperative tasks. Sun et al. compared the effectiveness of expert and novice teachers in collaborating with students to investigate the neural mechanisms that underlie the differences [13]. The study involved a task in which teachers drew a diamond shape without verbal communication, both independently and in collaboration with students. The levels of PT and empathy were also evaluated. Results indicated that expert teachers exhibited stronger ToM than novice teachers. Furthermore, fNIRS showed that expert teacher–student (ET-S) pairs had significant IBS in the frontal polar region and the rDLPFC during cooperative conditions. The brain–behavior correlation analysis revealed that IBS in the ET-S group correlated with teacher–student cooperation and ToM of expert teachers. The findings support the notion that teachers with extensive social experience possess better ToM, are more capable of empathizing with students, and can accurately predict their behavior.

The aforementioned study indicated that cooperation between expert teachers and students during joint action tasks is more effective than that of novice teachers, leading to increased IBS. However, it is unclear whether this IBS is solely due to continuous keystrokes. To further investigate, Sun et al. instructed teachers with varying levels of experience to conduct mathematical calculations independently and in cooperation with students [65]. Results showed that novice teacher–student (NT-S) dyads achieved higher accuracy in the cooperation condition than expert teacher–student (ET-S) dyads. However, expert teachers demonstrated higher levels of empathy than novice teachers, and showed increased IBS in the DLPFC with students. The DLPFC is implicated in integrating information about oneself and others [66,67], empathy, and inferring the intentions of others [68]. The detected brain regions in the study, which overlap with regions related to cognitive empathy and ToM [69], suggest that when expert teachers collaborate with students, they take the students’ perspective into consideration to infer their intentions.

## 5. A Potential Model of Empathy during Teacher–Student Interaction

**Figure 1:**
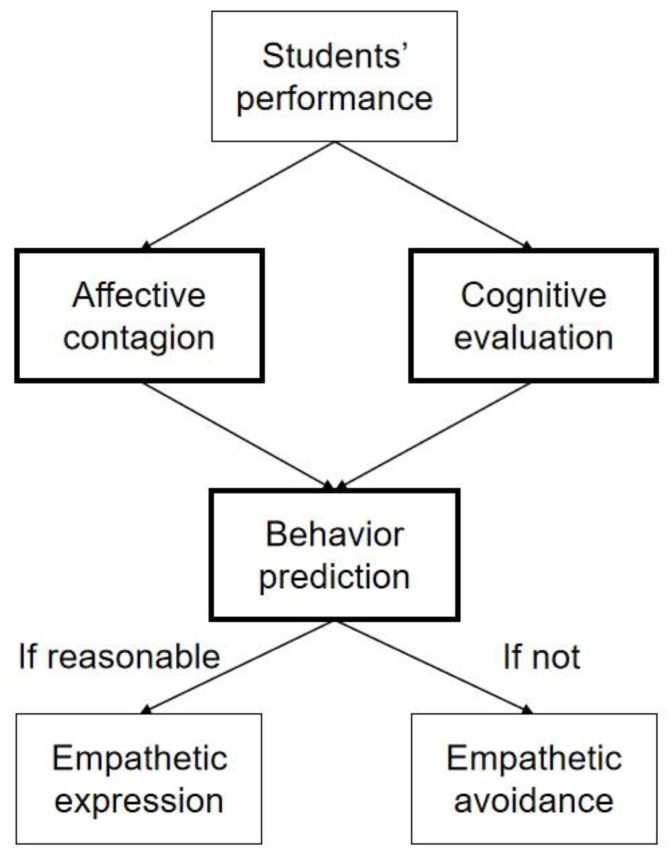
A potential model of empathy that integrates the affective contagion, cognitive evaluation, and behavior prediction aspects of teacher–student interactions.

In order for teacher–student interaction to function as a multilevel system, a dynamic mechanism of teacher empathy is required. In this study, we have formulated a potential empathy model for teacher–student interactions by integrating and building on previous research [21,22,23,25]. The proposed model consists of three interrelated components, namely affective contagion, cognitive evaluation, cognitive evaluation, and behavior prediction (empathetic behavior). This model not only incorporates the automatic processing of students’ behavior, but also includes controlled processing for adjustment and prediction of the teacher’s own empathy expression. As depicted in Figure 1, when teachers perceive sounds, movements, and expressions of their students, teachers’ affective and cognitive systems are triggered simultaneously. The activation of the MNS in the brain automatically causes the teacher to experience similar emotions to the students, leading to the development of affective empathy [28]. In parallel, several cortical regions such as mPFC, STS, TPJ, and TP may be stimulated to generate cognitive empathy, facilitating an evaluation of students’ emotional types, intensity, and underlying causes [32,33,34]. This notion was in accordance with a previous study showing that affective contagion and cognitive evaluation would be triggered simultaneously before empathy expression [70]. Next, before expressing empathy towards their students, teachers may choose to conduct a psychological evaluation to predict how their empathy expression may affect the students. The prediction–transmission hypothesis suggests that, teachers will first try to predict the knowledge level of their students to optimize their teaching approach [71], and this could be accompanied by increased activation of the teachers’ medial orbitofrontal cortex and ventral striatum [72]. This heightened brain activity prompts teachers to assess the appropriateness of their empathetic response. In situations in which a teacher judges their display of empathy to be appropriate, they will demonstrate observable behavioral indications of empathy. Conversely, if they feel that exhibiting empathy would be impractical, they might opt to refrain from empathizing with their students. This is because empathizing with others requires cognitive effort, and in challenging circumstances, individuals may choose to avoid empathizing altogether [73]. Similarly, teachers may decide to avoid empathizing in certain scenarios, based on their assessment of the potential outcomes of expressing empathy.

The ternary empathy model can be influenced by various social factors [74,75,76,77,78]. For example, as a teacher’s expertise grows, they become better equipped to quickly discern their students’ emotions, resulting in a more positive teaching environment and improved learning outcomes. Additionally, social factors, such as perceived similarity and familiarity [74,75], also play a role in this ternary empathy model.

Our ternary model integrates the affective component, including the MNS and Shared Intentionality Theory, along with the cognitive component, such as ToM and PT. It also incorporates the predictive component, allowing teachers to evaluate the appropriateness of their empathetic expression. By accurately reflecting the psychological process of teacher empathy, this model provides a strong foundation for future research aimed at enhancing the effectiveness of teacher–student interactions. To ensure the scientific and rational nature of the ternary empathy model developed from existing theories, it is crucial to perform additional verification.

There are some limitations to this model. Firstly, the model remains largely hypothetical and requires subsequent empirical testing to validate its scientific and rational nature, as well as to refine it. Secondly, the model only looks at the impact of empathy on teacher–student interaction and does not explore the reciprocal influence of the two factors. Going forward, it would be valuable to expand the theoretical model to explore this two-way influence, possibly through implementing a pretest-posttest design to investigate how this interaction subsequently improves empathy.

## 6. Future Directions

We identify two important avenues for future research. First, most existing research on teacher–student interaction focuses on the influence of teachers on students, neglecting the impact of students’ behavioral responses on teachers, despite it being a continuous and mutually influential process. For the sake of advancing teachers’ professional development, future research should account for both sides of the interaction. Moreover, it is essential to investigate interpersonal synchronization in special needs students, such as those with autism spectrum disorder (ASD) and attention-deficit hyperactivity disorder (ADHD). By comparing IBS between individuals with and without ASD or ADHD, we may pinpoint the brain regions responsible for cognitive impairments, and develop treatment plans based on scientific evidence. Teacher–student interaction extends beyond traditional classroom settings, including extracurricular activities, student-to-teacher questioning, and its impact on group cooperation. Research should explore these diverse forms of interaction to gain a more comprehensive understanding and provide practical insights for effective teaching.

Second, educators currently rely on single modal data for teaching and learning assessment, which overlooks important aspects of teaching such as nonverbal communication. To address this limitation, various multimodal technologies can be used to analyze teacher–student interactions, including EEG and fNIRS to observe changes in the cerebral cortex, eye tracking to monitor eye movements, skin conductance to examine physiological mechanisms, and video-based computer vision to detect teacher–student movement [79]. Such technologies enable a more comprehensive assessment of cognitive processes and neural responses during teacher–student interactions. Moreover, longitudinal research designs should be used to monitor changes in participants over time and across different settings to gain a better understanding of the development of interactions between teachers and students. This approach can provide a more comprehensive assessment of teaching effectiveness as a long-term process.

## 7. Conclusions

This study represents a preliminary attempt to parse the mechanisms of teacher empathy in teacher–student interaction, shedding new light on the process of teacher–student interaction. Importantly, building on previous research, we proposed a potential ternary model for empathy during teacher–student interactions.

## Data Availability

Not applicable.
